# Parental Acculturation and Its Effect on Preschool-Aged Children’s Health Behaviors Among Latinos in Nevada: A Cross-Sectional Study

**DOI:** 10.3390/nu16213610

**Published:** 2024-10-24

**Authors:** Christopher Johansen, Miguel Antonio Fudolig, Liliana Davalos, Brisa Rodriguez Alcantar

**Affiliations:** 1 Department of Social and Behavioral Health, School of Public Health, University of Nevada, Las Vegas (UNLV), Las Vegas, NV 89119, USA; davalosl@unlv.nevada.edu (L.D.); rodrib36@unlv.nevada.edu (B.R.A.); 2 Department of Epidemiology and Biostatistics, School of Public Health, University of Nevada, Las Vegas (UNLV), Las Vegas, NV 89119, USA; miguel.fudolig@unlv.edu

**Keywords:** acculturation, body mass index, early childhood, Latinos, obesity, physical activity, sugar-sweetened beverages

## Abstract

Background: Latino children in the United States (US) have a higher prevalence of overweight/obesity compared to white children. Previous studies suggest that acculturation to the US is associated with health behaviors such as diet, body mass index (BMI), and physical activity. However, the role of parental acculturation remains understudied, particularly with the use of validated measures. Objective: The objective of this study was to evaluate parental acculturation and its association with parental interpersonal factors and health behaviors in the preschool-aged child. Methods: Data were analyzed from 187 Latino parents in Nevada. Parents completed a self-reported, cross-sectional survey. Acculturation was assessed using Norris’ 4-item validated acculturation measure. The average age of the preschool-aged children was 45.5 months, and their mean BMI percentile was 96.4% (SD ± 18.7). The mean parental acculturation score was 2.1 (SD ± 1.2). Children were physically active an average of 4.9 (SD ± 2.0) days per week. After controlling for covariates, the results indicated that parental acculturation was positively associated with physical activity and sugar-sweetened beverage consumption. However, parental acculturation was not associated with child BMI percentile, or the consumption of fruits, vegetables, and sweet snacks. Conclusions: These findings can inform future research on culturally tailored intervention strategies to boost physical activity and reduce sugar-sweetened beverage intake among Latino preschool-aged children.

## 1. Introduction

In the United States (US), Hispanics/Latinos are the largest and fastest-growing ethnic minority group [1,2]. Latinos suffer significant health disparities compared to other racial and ethnic groups [3,4], such as higher rates of diabetes, hypertension, non-alcoholic fatty liver disease, cardiovascular disease, and obesity [5,6,7,8,9]. These disparities are also observed among Latino children aged 2–5 years [10]. For example, Latino children are disproportionately affected by obesity [11,12]. By preschool age (2–5 years old), Latino children are three times more likely to be obese compared to non-Latino white children [12], and approximately one-and-a-half times more likely to be obese than children from Mexico [13]. Policymakers should support evidence-based interventions aimed at preventing the onset of obesity among Latinos during early childhood. Preventing obesity in childhood can reduce the risk of adult comorbidities such as cardiovascular disease, diabetes, and cancer [14]. Therefore, it is critical to understand the determinants of Latino childhood obesity, enabling public health practitioners to intervene with Latino parents and their children to prevent the onset of obesity in early childhood.

A potential determinant of obesity among Latinos is acculturation, the dual process of cultural and psychological change that takes place as a result of contact between two or more cultural groups and their individual members [15]. Prior evidence from studies on Latino adults and adolescents suggests that being born in the US and having a longer duration of residence in the US are associated with a higher prevalence of obesity [16,17,18], suggesting that acculturation may influence obesity. Preschool-aged children depend on their parents for their needs, including nutrition and physical activity; however, few studies have examined the influence of parental acculturation on a preschool-aged children’s health behaviors. Recent evidence suggests that parental acculturation may influence a child’s weight status and body mass index (BMI) [19]. Prior studies that have examined this association suggest that acculturation is positively associated with the dietary behaviors [20,21,22] and physical activity [23,24] of school-aged children and adolescents, but these relationships have not been extensively studied among preschool-aged children [25,26].

There is a need to understand the role of acculturation in obesity, as well as other contributors to the obesity epidemic, such as nutrition and physical activity [9,27,28,29,30,31]. For example, previous studies suggest that acculturation is negatively associated with fruit and vegetable consumption [32,33]. Acculturation has been positively associated with physical activity among Latino adolescents and children [28,34]. There is a dearth of research on the acculturation of parents and its association with diet and physical activity in preschoolers. A prior study among Latino parents of preschoolers indicated that while Latino parents know their children should eat healthy foods and be physically active, they lack the confidence/self-efficacy to role model these behaviors and create environments supportive of recommended behaviors [35,36]; however, these studies did not measure acculturation.

Obesity among Latino children is also a regional health concern in Nevada, where recent studies suggest that 23.9% of Latino children are obese, compared to the national average of 13.7% [37,38]. This underscores the importance of identifying potential targets for intervention, with acculturation potentially playing a role in such a disparity. Therefore, the current study aimed to address existing research gaps by examining the effect of parental acculturation on their preschool-aged children’s nutrition, physical activity, and obesity. It was hypothesized that parental acculturation to the US would be positively associated with children’s BMI percentile; parental acculturation to the US would be positively associated with children’s physical activity; and parental acculturation to the US would be negatively associated with children’s fruit/vegetable consumption, and positively associated with their consumption of sugar-sweetened beverages and sweet snacks.

## 2. Materials and Methods

### 2.1. Study Design

Primary data collection occurred from October 2022 to March 2024. This cross-sectional pilot study employed convenience sampling and snowball methods to recruit participants. Parents of preschool-aged children were recruited at free mobile clinics, health fairs, and other community events. The ethical standards recognized by the Declaration of Helsinki were followed. The research protocol of the study was approved by the Institutional Review Board of the University of Nevada, Las Vegas (IRB Protocol #UNLV 2022-502). Parents provided verbal informed consent. Spanish versions of the consent form were available for parents who required them.

### 2.2. Procedures and Participants

Researchers partnered with Research, Education and Access to Community Health (REACH), a local non-profit organization that provides free services and referrals to the Hispanic/Latino community in Nevada. Participants were recruited and screened at various REACH events and other local community events. Parents were eligible to participate if they met the inclusion criteria: (1) parent self-identified as Hispanic/Latino, (2) was at least 18 years of age or older, (3) had at least one child 2–5 years of age, (4) was living in Nevada. Assessments included parent-reported height and weight measurement and a brief questionnaire covering health and dietary habits (e.g., acculturation, BMI, nutrition, physical activity). Participants received a $10 Visa Card for their participation. In total, 256 caregivers completed the survey; however, 69 respondents were removed due to ineligibility, i.e., did not identify as Hispanic/Latino, child was 6 years or older, caregiver was not a parent (e.g., grandparent, aunt, uncle, nanny, etc.). The final sample was *n* = 187 respondents.

### 2.3. Measures

#### 2.3.1. Acculturation

Parental acculturation was assessed with Norris’ 4-item validated Brief Acculturation Scale for Hispanics (BASH) [39], including items such as “What language do you usually speak at home?” with respondents indicating their preference using one of the five coded response options: “Only Spanish” coded as 1, “More Spanish than English” coded as 2, “Both English and Spanish equally” coded as 3, “More English than Spanish” coded as 4, and “Only English” coded as 5. The mean score was calculated on the four items with scores ranging from 1–5, with higher scores reflecting greater acculturation to the US. The BASH has good internal consistency with a reported Cronbach alpha of $\alpha_{C}$ = 0.90 [39].

#### 2.3.2. BMI Percentile

Parent-reported measurements of their child (height, weight) were used to calculate body mass index percentile using obesity classification based on the Centers for Disease Control and Prevention (CDC) growth chart norms-based BMI percentile calculator for child age and gender [40]. BMI percentile was used as a continuous variable in data analysis.

#### 2.3.3. Physical Activity

Physical activity was assessed with one item: “During the past 7 days, on how many days was your child physically active for a total of at least 60 min per day? Not including while in school, add up all the time s/he spent doing any kind of physical activity that increased her/his heart rate and made her/him breathe hard at least some of the time.” Response options ranged from 0–7 [41].

#### 2.3.4. Sugar-Sweetened Beverages

The NCI’s Dietary Screening Questionnaire (DSQ) was adapted for parent-reported nutrition intake for their child [42,43]. A sum score was calculated based on 4 items: flavored milk, sweetened drinks (KoolAid, lemonade, Sunny D, aguas frescas, Caprisun), sports drinks (Gatorade, Powerade), and regular soda. Response options were coded as follows: “Never” coded as 0, “Several times per month” coded as 1, “Several times per week” coded as 2, “Once every day” coded as 3, and “More than once per day” coded as 4.

#### 2.3.5. Sweet Snacks

A sum score of 4 items of the DSQ was used to measure sweet snack consumption. Respondents answered the following questions: “How often does your child eat doughnuts, sweet rolls, Danish, muffins, pan dulce, or Pop Tarts?“ “How often does your child eat ice cream or other frozen desserts?” “How often does your child eat cookies, cake, pie, or brownies?” and “How often does your child eat chocolate or any other types of candy?” Response options were coded as follows: “Never” coded as 0, “Several times per month” coded as 1, “Several times per week” coded as 2, “Once every day” coded as 3, and “More than once per day” coded as 4 [43].

#### 2.3.6. Fruits

A brief validated 1-item measure of fruit consumption [44] assessed children’s fruit intake by asking parents, “How often does your child eat fruit? Include fresh, frozen, or canned fruit. Do not include juices.” Response options ranged from “Never” coded as 0, “Several times per month” coded as 1, “Several times per week” coded as 2, “Once every day” coded as 3, to “More than once per day” coded as 4.

#### 2.3.7. Vegetables

A brief validated 1-item measure to assess vegetable consumption was used [44]. Parents were asked, “How often does your child eat vegetables? Do not count potatoes, corn, or beans.” Response options ranged from “Never” coded as 0, “Several times per month” coded as 1, “Several times per week” coded as 2, “Once every day” coded as 3, to “More than once per day” coded as 4 [44].

#### 2.3.8. Covariates

Parent age, parent education, and household income were included as covariates.

### 2.4. Statistical Analyses

Data were analyzed using R software (version 4.4.1). Descriptive statistics for all measures were included: acculturation, nutrition consumption, physical activity, child BMI percentile, and covariates. Cronbach’s alpha was computed for the acculturation, sweet snack consumption, and sugar-sweetened beverage consumption scales to measure the internal consistency of the scales. Confidence intervals for the Cronbach’s alpha were estimated using bootstrapping methods. Confirmatory factor analysis (CFA) was used to validate a three-factor structure that included parental acculturation, sweet snack consumption, and sugar-sweetened beverage consumption as latent variables. The goodness of model fit was assessed using Hu and Bentler’s [45] recommended thresholds for the comparative fit index (CFI), root mean square error of approximation (RMSEA), and the standardized root mean square residual (SRMR).

Data were analyzed using regression models. Linear regression models were used to analyze the association between parental acculturation and children’s BMI percentage, physical activity, and the sum scores for sweet snacks and sugar-sweetened beverage consumption. Linearity, homoscedasticity, and normality of residuals were tested using model diagnostic methods. Fruit and vegetable consumption were individually treated as ordinal variables and analyzed using the proportional odds model. Parental age, income, and educational attainment were added to the model as covariates. For child BMI percentile, 41 responses were excluded due to extraneous BMI estimates of parent self-reporting; therefore, the sample was restricted to include data points with BMI z-scores between −6 and 6. The significance level was set to 0.05 for all hypothesis tests.

### 2.5. Post-Hoc Power Analysis

A post-hoc power analysis was conducted. Results imply that the multiple linear regression analysis can detect a small to medium effect size ($Cohen^{’}s\;f^{2}=0.05$) with 86.0% power at a significance level of 0.05. The study′s design was therefore robust enough to detect statistically significant associations.

## 3. Results

### 3.1. Participant Characteristics and Correlation Analyses

The descriptive statistics of participants are summarized in Table 1. All mean scores are presented in a Mean ± SD format. The mean parental acculturation score was 2.1 ± 1.2 (Range: 1–5). The mean child BMI percentile was 96.4% ± 18.7, mean age of child was 45.5 months ± 15.4, and 50.3% of the children were boys. Parents reported children were physically active an average of 4.9 days per week. Children′s mean fruit consumption was 3.8 ± 1.1 (range 1–5), and mean vegetable consumption was 2.6 ± 1.0 (Range 1–5). Nearly a third of children (32.1%) were healthy weight (5th to <85th percentile), 24.1% were overweight (85th to <95th percentile), and 43.8% were obese (>95th percentile). Approximately 70% of parents had a high school diploma or less. Over half of parents (51.9%) were married. Approximately 40% of parents reported a total household annual income less than $30,000 per year. Table 2 displays the strength of association between the dependent variables considered in this study. Fruit and vegetable consumption frequencies were found to be positively associated, with an estimated Spearman correlation coefficient of r = 0.69 (*p* < 0.001). However, they were not associated with the consumption of sweet snacks or sugar-sweetened beverages. Consumption of sweet snacks was positively correlated with sugar-sweetened beverages, with an estimated Spearman correlation coefficient of r = 0.41 (*p* < 0.001). A notable result from this correlation analysis is the lack of association between consumption frequency and the BMI% of the children. The Spearman correlation coefficients between the BMI% and the frequencies of fruit, vegetable, sweet snack, and sweet beverage consumption were estimated to be −0.08 (*p* = 0.33), −0.06 (*p* = 0.45), −0.05 (*p* = 0.57), and −0.01 (*p* = 0.89), respectively.

### 3.2. Internal Consistency and Factor Structure Validation

There was acceptable internal consistency for the 4-item BASH acculturation scale ($\alpha_{C}$ = 0.95, 95% CI: 0.94–0.96), 4-item sweet snack consumption scale ($\alpha_{C}$ = 0.83, 95% CI: 0.75–0.88), and the 4-item sweet beverage consumption scale ($\alpha_{C}$ = 0.69, 95% CI: 0.56, 0.78). The 3-factor structure yielded the following robust fit index values: CFI = 0.97 (>0.95), RMSEA = 0.07 (<0.08), and SRMR = 0.07 (<0.08), which implies a good fit with the data.

### 3.3. Regression Analyses

Table 3 displays the unstandardized and standardized coefficients from the multiple linear regression models. Four child outcomes were modeled. Model 1 suggests that parental acculturation was not associated with BMI percentile (standardized B = –0.01, *p* = 0.92). Model 2 suggests that parental acculturation was positively associated with the reported days of physical activity in children (standardized B = 0.31, *p* = 0.004). Specifically, for every 1-standard deviation increase in parental acculturation, there was an increase of 0.31 standard deviation units of physical activity per week. Model 3 suggests that parental acculturation was not associated with the consumption of sweet snacks (standardized B = 0.07, *p* = 0.46). Model 4 indicates that parental acculturation was positively associated with the consumption of sugar-sweetened beverages by children (standardized B = 0.29, *p* = 0.006). A 1-standard deviation increase in parental acculturation corresponded to an increase of 0.29 standard deviation units of a child’s total sugar-sweetened beverage consumption score.

As shown in Table 4, an ordinal logistic regression analysis based on a proportional odds model found no evidence of any effect of parental acculturation on fruit consumption (adjusted OR = 1.20, *p* = 0.46) or vegetable consumption (adjusted OR = 1.32, *p* = 0.09). However, in the study sample, those who had some college education were 2.35 times more likely to have their child consume fruit compared to those who had high school education or lower (*p* = 0.02).

## 4. Discussion

The objective of this study was to evaluate parental acculturation and its association with child BMI percentile, nutrition, and physical activity. More acculturated parents may contribute to their child’s increased physical activity, as well as the child’s higher consumption of sugar-sweetened beverages. However, parental acculturation was not associated with child BMI percentile, fruit, vegetable, or sweet snack consumption. The finding regarding educational attainment highlights the role of parental education in promoting healthier fruit consumption.

Parental acculturation was positively associated with children’s sugar-sweetened beverage consumption, as predicted. These findings align with previous studies among children and adolescents and extend these associations to preschool-aged children [25]. Parental acculturation may influence dietary habits, including the consumption of sugar-sweetened beverages of their preschool-aged children. As parents become more acculturated, they often adopt dietary patterns and preferences from the dominant, i.e., “US” culture, which may include higher consumption of sugar-sweetened beverages. This shift can be attributed to increased exposure to, and marketing of, processed foods prevalent in the dominant culture [46]. As parents become more acculturated, their dietary practices, including those related to sugar-sweetened beverages, might change, potentially impacting their children’s intake. Greater acculturation to the US has been associated with increased sugar-sweetened beverage consumption due to changes in dietary norms and the increased availability of sugary products [47]. Parental acculturation is an important factor to consider when addressing children’s consumption of sugar-sweetened beverages.

Parental acculturation was positively associated with physical activity, as predicted. These findings corroborate those from other studies [23,24] and extend these associations to preschool-aged children. As parents become more acculturated, their attitudes and practices regarding physical activity may change, which, in turn, affects their children′s activity levels. It is possible that children are more physically active in the US and acculturation in this context is a potential protective factor. The ‘healthy immigrant paradox’ [20] posits that, as individuals acculturate, they adopt unhealthy behaviors/habits the longer they are in the US; however, increased physical activity may represent a positive aspect of the acculturation process. Parental acculturation can influence family routines, and the value placed on physical activity can be beneficial to preschool-aged children’s health.

The lack of association between parental acculturation and a child′s fruit, vegetable, and sweet snack consumption is notable, and differs from studies that have shown associations among these outcomes [32,33]. Parental acculturation also was not significantly associated with child BMI percentile. Two previous studies among Latino children and adolescents have also not detected this association [48,49]. It is possible that, in early childhood, parents exert more control over their preschool-aged child’s dietary intake, thus acculturation may not influence these outcomes among young children compared to previously established associations among other age groups of Latinos.

### 4.1. Implications and Future Directions

Our findings underscore the role of acculturation to the US in shaping children’s health behaviors, such as increased physical activity and increased sugar-sweetened beverage intake. These findings have implications for public health research. Researchers should continue to assess acculturation status using validated measures. There have been clinical interventions to reduce sugar-sweetened beverages among young children that show promise [50]. For example, Beck and colleagues found that a brief 20 min educational intervention on healthy beverage consumption for Latino parents led to a 4-ounce reduction in their 2-year-old children’s consumption of 100% fruit juice at a 2-week follow-up [50].

Culturally tailoring educational and behavioral strategies to different levels of acculturation might enhance the effectiveness of these interventions. For instance, describing foods and beverages predominant in the Hispanic/Latino traditional diet (e.g., *aguas frescas*) may have a stronger impact than simply providing examples of typical “US” beverages. Previous culturally tailored interventions have shown to decrease obesogenic behaviors but have not fully considered acculturation [51,52]. Interventions that address how acculturation affects dietary choices could help parents make healthier beverage choices and understand the impact of sugar-sweetened beverages on health. Culturally tailored interventions designed for Latino parents could provide bilingual materials (English and Spanish) that explain the health risks of sugar-sweetened beverages (e.g., sodas, flavored drinks) and offer alternatives like water or homemade *aguas frescas* without added sugar. This takes into consideration traditional Latino beverages that families might maintain from their Latino culture while also addressing the often unhealthy habits in the US. This proposed strategy could help Latino parents to better understand how beverage choices differ between their Latino culture and US norms, making them more likely to offer healthier alternatives like water or milk instead of sugar-sweetened beverages.

Future interventions should focus on home-based and/or school-based strategies to increase physical activity among less-acculturated households. It is possible that lay community members (i.e., *promotoras*/community health workers) can deliver these interventions in community settings, e.g., schools. These community health workers, being from the same communities and sharing similar lived experiences as the participants, may have stronger rapport with them and can enhance the delivery of the intervention. Offering free or low-cost family-style physical activity classes led by *promotoras* (such as walking or dancing) at local parks, community recreation centers, and/or faith-based organizations might help less-acculturated parents and their young children adopt and sustain more active lifestyles.

The findings from this study highlight the importance of integrating cultural factors into strategies that promote physical activity and decrease sugar-sweetened beverage consumption. For instance, community programs could be developed to support and encourage active lifestyles in ways that align with the cultural values and practices of more acculturated families. Educating parents about the benefits of physical activity and how their own acculturation might influence their children’s activity levels could help increase awareness and encourage more active behaviors within families.

Future research should attempt to identify the causal mechanisms behind these associations to provide deeper insights into how cultural factors shape dietary habits. Elucidating the linkages between parental acculturation and dietary intake and physical activity outcomes via longitudinal cohort studies is warranted. Additionally, researchers should use objective measures to assess anthropometrics and nutrition intake.

### 4.2. Strengths and Limitations

This study has a few limitations that must be considered. First, this study relied on a convenience sample; therefore, this sample may not be fully representative of Latinos (parents and their young children) in Nevada. Another limitation is the relatively small sample size, but the current study was sufficiently powered (86%). The study analyzed cross-sectional data, which prevents establishing a causal relationship between parental acculturation and obesity outcomes in their children. Another limitation is self-reported measures to determine health behaviors, which are susceptible to recall bias.

This study also includes several notable strengths. To our knowledge, this study is the first to explore the association of parental acculturation and child health behaviors in Nevada. In fact, this study brings needed attention to an understudied Latino population. Another strength is the evaluation of parental acculturation using a validated acculturation measure, which few studies have utilized.

## 5. Conclusions

In summary, Latino parents with greater acculturation to the US reported that their preschool-aged children engaged in increased physical activity but also consumed more sugar-sweetened beverages. This reflects the complex interplay between adopting new cultural practices, environmental influences, and lifestyle changes. While acculturation can lead to healthier behaviors such as increased physical activity, it can also expose individuals to dietary patterns prevalent in the new culture, which may include a higher intake of sugar-sweetened beverages. Overall, the significant associations identified in this study indicate that parental acculturation plays a crucial role in shaping children′s physical activity and sugar-sweetened beverage consumption. Therefore, interventions and policies aimed at promoting physical activity and reducing sugar-sweetened beverage consumption should take cultural and acculturation factors into account to enhance their effectiveness.

## Figures and Tables

**Table 1:** Descriptive statistics of participants.

Parent Characteristics	Frequency	*n* (%)	Mean ± SD ^b^
Relation to Child	Mother Father	170 (90.9%) 17 (9.1%)	
Parent Age			33.4 ± 6.5
Educational Attainment	8th grade or less	43 (23.0%)	
	Some high school	32 (17.1%)	
	High school graduate or GED	49 (26.2%)	
	Some college or 2-year degree	29 (15.5%)	
	4-year college degree	26 (13.9%)	
	Graduate Degree	6 (3.2%)	
	Missing	2 (1.1%)	
Marital Status	Divorced/Separated	16 (8.6%)	
	Living with partner	30 (16.0%)	
	Married	97 (51.9%)	
	Single	41 (21.9%)	
	Widowed	1 (0.5%)	
	Missing	2 (1.1%)	
Total Household Annual Income	Less than $20,000	49 (26.2%)	
	$20,000–$30,000	27 (14.4%)	
	$30,001–$50,000	37 (19.8%)	
	$50,001–$75,000	22 (11.8%)	
	More than $75k	12 (6.4%)	
	Prefer not to answer/Missing	40 (21.4%)	
Parental Acculturation			2.1 ±1.2
**Child Characteristics**	**Frequency**	***n* (%)**	**Mean ± SD ^b^**
Child Age (Months)			45.5 ± 15.4
Child Sex	Male	94 (50.3%)	
	Female	84 (44.9%)	
	Missing	9 (4.8%)	
Child Physical Activity (days)			4.9 ± 2.0
Child Fruit Consumption Score			3.8 ± 1.1
Child Vegetable Consumption Score			2.6 ± 1.0
Child Sweet Snack Consumption			8.5 ± 2.8
Child Sugar-Sweetened Beverage Consumption Score			7.3 ±2.7
Child BMI%			96.4 ±18.7
Child BMI Classifier ^a^	Healthy Weight	60 (32.1)	
	Overweight	45 (24.1)	
	Obese	85 (43.8)	

Note: ^a^ = BMI for sex and age classified as: ‘healthy weight’ with BMI% 5% to <85%; ‘overweight’ with BMI% 85% to <95%; and ‘obese’ with BMI% <95%; ^b^ = Standard Deviation.

**Table 2:** Spearman correlation coefficients and corresponding *p*-values (in parentheses) of the response variables considered for analysis.

	BMI%	Fruit Consumption	Vegetable Consumption	Sweet Snacks	Sugar-Sweetened Beverages
BMI%	1.00	−0.08 (0.33)	−0.06 (0.45)	−0.05 (0.57)	−0.1 (0.89)
Fruit Consumption		1.00	0.69 (<0.001)	−0.01 (0.95)	−0.01 (0.89)
Vegetable Consumption			1.00	−0.01 (0.93)	−0.06 (0.45)
Sweet Snacks				1.00	0.41 (<0.001)
Sugar-Sweetened Beverages					1.00

**Table 3:** Multiple linear regression of child behaviors regressed on parental acculturation and covariates.

Unstandardized B	Model 1 BMI%	Model 2 Physical Activity	Model 3 Sweet Snacks	Model 4 Sugar-Sweetened Beverages
Intercept	94.7 ± 12.3 * (70.3, 119.1)	5.71 ± 1.13 * (3.47, 7.95)	8.84 ± 1.49 * (5.88, 11.79)	7.64 ± 1.56 * (4.56, 10.72)
Acculturation	−0.02 ± 1.81 (−3.62, 3.58)	0.52 ±.18 ** (0.17, 0.87)	0.17 ± 0.23 (−0.29, 0.64)	0.68 ± 0.24 ** (0.19, 1.16)
Parent Age	0.06 ± 0.33 (−0.59, 0.70)	−0.05 ± 0.03 (−0.11, 0.01)	−0.02 ± 0.04 (−0.10, 0.06)	−0.05 ± 0.04 (−0.13, 0.03)
Income (≥$30k)	0.63 ± 3.93 (−7.17, 8.43)	−0.62 ± 0.39 (−1.40, 0.15)	−0.04 ± 0.52 (−1.08, 0.99)	0.03 ± 0.55 (−1.05, 1.11)
Education (>High School)	−0.97 ± 3.95 (−8.79, 6.86)	0.17 ± 0.38 (−0.57, 0.92)	−0.42 ± 0.50 (−1.42, 0.57)	−0.42 ± 0.52 (−1.46, 0.62)
**Standardized** **B**	**Model 1** **BMI%**	**Model 2** **Physical Activity**	**Model 3** **Sweet Snacks**	**Model 4** **Sugar-Sweetened Beverages**
Acculturation	−0.001 ± 0.116 (−0.230, 0.228)	0.308 ± 0.104 ** (0.102, 0.513)	0.074 ± 0.099 (−0.122, 0.269)	0.290 ± 0.105 ** (0.083, 0.497)
Parent Age	0.018 ± 0.108 (−0.195, 0.232)	−0.174 ± 0.098 (−0.368, 0.020)	−0.042 ± 0.093 (−0.226, 0.142)	−0.122 ± 0.099 (−0.317, 0.073)
Income (≥$30k)	0.034 ± 0.210 (−0.383, 0.450)	−0.314± 0.196 (−0.702, 0.074)	−0.016 ± 0.188 (−0.389, 0.357)	0.012 ± 0.199 (−0.382, 0.407)
Education (>High School)	−0.052± 0.211 (−0.470, 0.367)	0.086± 0.190 (−0.289, 0.461)	−0.151 ± 0.181 (−0.510, 0.207)	−0.153 ± 0.192 (−0.532, 0.226)

Note: Coefficients are written as Estimate, Std. error for BMI%, Physical Activity, Sweet Snacks, and Sugar-Sweetened Beverages with 95% confidence intervals (in parentheses). * indicates statistical significance at the 0.05 level. ** indicates significance at the 0.01 level.

**Table 4:** Ordinal logistic regression analyses of child fruit and vegetable consumption regressed on parental acculturation and covariates.

	Model 5 Child Fruit Consumption	Model 6 Child Vegetable Consumption
Parental Acculturation	1.20 (0.88, 1.65)	1.32 (0.96,1.83)
Parent Age	1.03 (0.98,1.09)	1.04 (0.98, 1.10)
Income (≥$30k)	1.00 (0.50, 2.02)	0.59 (0.29, 1.19)
Education (>High School)	2.35 (1.17, 4.77) *	1.84 (0.92, 3.72)

Note: Estimated odds ratios of the ordinal logistic regression analysis for fruit and vegetable consumption. * denotes significance at the 0.05 level. The 95% confidence limits are enclosed in parentheses.

## Data Availability

Data and R code are available by request.
